# Hydrogen Peroxide Generation as an Underlying Response to High Extracellular Inorganic Phosphate (Pi) in Breast Cancer Cells

**DOI:** 10.3390/ijms221810096

**Published:** 2021-09-18

**Authors:** Marco Antonio Lacerda-Abreu, Thais Russo-Abrahão, Nathália Rocco-Machado, Daniela Cosentino-Gomes, Claudia Fernanda Dick, Luiz Fernando Carvalho-Kelly, Michelle Tanny Cunha Nascimento, Thaís Cristino Rocha-Vieira, José Roberto Meyer-Fernandes

**Affiliations:** 1 Instituto de Bioquímica Médica Leopoldo de Meis, Universidade Federal do Rio de Janeiro, Rio de Janeiro 21941-901, RJ, Brazil; marcoantoniolacerdaabreu@gmail.com (M.A.L.-A.); trabrahao@gmail.com (T.R.-A.); rocconathalia@gmail.com (N.R.-M.); danicosentino@yahoo.com.br (D.C.-G.); claudia.dick@gmail.com (C.F.D.); lfernandock@gmail.com (L.F.C.-K.); michelletanny@yahoo.com.br (M.T.C.N.); thaiscrvieira@outlook.com (T.C.R.-V.); 2 Instituto Nacional de Ciência e Tecnologia em Biologia Estrutural e Bioimagem, Rio de Janeiro 21941-590, RJ, Brazil; 3 National Heart, Lung, and Blood Institute, NIH, Bethesda, Rockville, MD 20814, USA; 4 Departamento de Bioquímica, Universidade Federal Rural do Rio de Janeiro, Seropédica 23890-000, RJ, Brazil; 5 Instituto de Biofísica Carlos Chagas Filho, Universidade Federal do Rio de Janeiro, Rio de Janeiro 21941-901, RJ, Brazil

**Keywords:** Pi-induced H_2_O_2_ production, breast cancer cells, H^+^-dependent Pi transport, Na^+^-dependent Pi transport, cell migration

## Abstract

According to the growth rate hypothesis (GRH), tumour cells have high inorganic phosphate (Pi) demands due to accelerated proliferation. Compared to healthy individuals, cancer patients present with a nearly 2.5-fold higher Pi serum concentration. In this work, we show that an increasing concentration of Pi had the opposite effect on Pi-transporters only in MDA-MB-231 when compared to other breast cell lines: MCF-7 or MCF10-A (non-tumoural breast cell line). Here, we show for the first time that high extracellular Pi concentration mediates ROS production in TNBC (MDA-MB-231). After a short-time exposure (1 h), Pi hyperpolarizes the mitochondrial membrane, increases mitochondrial ROS generation, impairs oxygen (O_2_) consumption and increases PKC activity. However, after 24 h Pi-exposure, the source of H_2_O_2_ seems to shift from mitochondria to an NADPH oxidase enzyme (NOX), through activation of PKC by H_2_O_2_. Exogenous-added H_2_O_2_ modulated Pi-transporters the same way as extracellular high Pi, which could be reversed by the addition of the antioxidant N-acetylcysteine (NAC). NAC was also able to abolish Pi-induced Epithelial-mesenchymal transition (EMT), migration and adhesion of MDA-MB-231. We believe that Pi transporters support part of the energy required for the metastatic processes stimulated by Pi and trigger Pi-induced H_2_O_2_ production as a signalling response to promote cell migration and adhesion.

## 1. Introduction

Breast cancer is a heterogeneous and complex disease that varies in morphological and molecular structure, as well as the clinical course, requiring different approaches to diagnosis and treatment [1,2]. Cell lines are classified based on their expression patterns of oncogenic receptors: oestrogen receptor (ER), progesterone receptor (PR) and human epidermal growth factor receptor type 2 (HER2), such as MCF-7 (Luminal A, ER^+^, PR^+^, and HER2^−^) or MDA-MB-231 (triple-negative expression: ER^−^, PR^−^ and HER2^−^) [2]. Triple-negative breast cancer cells (TNBC) are significantly more aggressive than tumours of the other molecular subtypes, mainly because the patients have substantially shorter survival following their first metastatic event [3]. In vitro studies have determined a higher metastatic capacity of MDA-MB-231 cells when compared to MCF-7 [4].

Cancer cells are self-sufficient in the signals that promote growth; however, there is evidence that cancer growth can be influenced by exogenous nutritional factors, including Pi [5]. Pi is an essential nutrient, being a constituent of phospholipids and nucleotides (RNA or DNA), and it is associated with energy metabolism, either in the form of ATP or in its free form as a substrate for the intermediates of metabolic pathways [6]. There is a consensus that tumour cells have high Pi demand due to accelerated proliferation, according to the growth rate hypothesis (GRH) [7]. In line with this, in cancer patients, serum Pi concentrations were shown to be above (2.52 ± 0.72 mmol/L) the regular values found in healthy individuals (1.09 ± 0.19 mmol/L) [8]. In addition, it is hypothesized that there is an increase in the amount of inorganic phosphate in the tumour microenvironment during inflammatory hyperaemia (increases in blood flow rate and blood volume) and hyperphosphatemia (caused by elevated serum levels of dysregulated inorganic phosphate) [9]. High levels of dietary Pi have been associated with the tumorigenesis of various types of cancer [10,11,12].

It has already been shown that metastasis establishes and forms secondary tumours at organs with a higher Pi content compared to the organ containing the original tumour [11]. Lung and breast cancer cells showed high migratory capacity through the expression of osteopontin regulated by the C2 protein of the Forkhead Box (FOXC2) after treatment with high concentrations of Pi (3 or 5 mM) [13]. In breast cancer cell lines MDA-MB-231 and MCF-7, it was shown that Pi acts as a novel signalling molecule capable of eliciting a robust antiproliferative action only in MDA-MB-231 breast cancer cells [14]. Additionally, Pi sharply increases doxorubicin-induced cytotoxicity in MDA-MB-231 cells by the induction of apoptosis [15].

Altered levels of reactive oxygen species (ROS) are typical hallmarks of cancer progression. The increased production of ROS is attributable to the activation of oncogenes (such as Ras, Bcr-Abl, and c-Myc) and/or inactivation of tumor suppressor genes (such as p53), resulting in aberrant metabolism and alteration in antioxidant production following oxidative damage [16,17]. Besides, it is demonstrated that the enhanced ROS production is associated with highly metastatic phenotype by the activation of PI3K/AKT, MAPK, RAS, and Necrosis Factor-kappa B (NF-kB) pathways [17,18]. In addition, ROS have been shown to induce epithelial-mesenchymal transition (EMT) in cancer cells [16], in which transformed epithelial cells acquire mesenchymal phenotype, including motility and invasiveness. During EMT, cells lose epithelial markers (E-cadherin, occludin, and cytokeratins) and begin to express mesenchymal markers (vimentin and fibronectin). EMT is regulated by several transcription factors, including Snail, Slug, and NF-κB [4].

The main cell ROS molecules, hydrogen peroxide (H_2_O_2_) and the superoxide anion radical (O_2_^−^), are key redox signalling agents generated by more than 40 enzymes, prominently including NADPH oxidases and the mitochondrial electron transport chain [19,20,21]. The mitochondrial electron transport chain leaks electrons from Complexes I, II, and III to molecular oxygen to generate superoxide, which is rapidly converted into H_2_O_2_. H_2_O_2_ has a particularly important role as a signalling molecule because of its membrane-diffusible nature and stability [21].

Some studies have shown that Pi increases ROS production. In isolated mitochondria, Pi is co-transported with H^+^ (or in exchange for OH^−^), which decreases ΔpH, causing a rapid and pronounced hyperpolarization of the electrical potential across the inner mitochondrial membrane (ΔΨm), which promotes an increase in ROS production [22,23,24,25,26,27,28]. However, a different Pi-induced ROS production pathway was observed in bovine aortic endothelial cells (BAECs) through PKC and NADPH oxidase (NOX) activation [29].

Pi enters cells via Na/Pi co-transporters. These co-transporters constitute two large families of Na^+^-dependent Pi transporters that have been characterized in mammals: SLC20 and SLC34. The SLC34 family consists of three members, namely, NaPi-IIa (SLC34A1), NaPi-IIb (SLC34A2), and NaPi-IIc (SLC34A3) [30]. In 2018, our group showed that the high-affinity Na^+^-dependent Pi transport activity is higher in MDA-MB-231 compared to other breast cancer cell lines (MCF-7 and T47-D). We observed that the inhibition of Na^+^-dependent Pi transport significantly reduced tumour cell migration and adhesion [31]. In 2019, using the same MDA-MB-231 cells, we characterized a low-affinity H^+^-dependent Pi transporter related to cell migration and adhesion processes, especially in the presence of high Pi concentrations [32]. A decrease in NaPi-IIb expression and Na^+^-dependent Pi transport in the presence of 2 mM Pi were shown [32], and as a compensatory mechanism, H^+^-dependent Pi transport was stimulated [33].

In non-cancer models, the correlation between Pi and increased ROS production is already known [22,23,24,25,26,27,28,29]. Nevertheless, even though there is an assumption that Pi is a required nutrient for cancer cell progression, it has never been linked to the generation of ROS. Thus, in this work, we evaluated whether Pi could induce H_2_O_2_ production in breast cancer cells and its possible role in migration and adhesion. Here, we used the non-tumoral breast cell line (MCF10-A), a luminal A cell line (MCF-7), and a TNBC cell line (MDA-MB-231) to investigate the effect of high extracellular Pi on Pi transport, H_2_O_2_ production, cell migration, and adhesion. Two types of Pi transport were evaluated at a high Pi: Na^+^-dependent and H^+^-dependent and possible regulatory mechanisms for Pi transporters.

## 2. Results

### 2.1. Effect of Increased Pi Concentrations on Pi Transport in Breast Cell Lines

**Figure 1:**
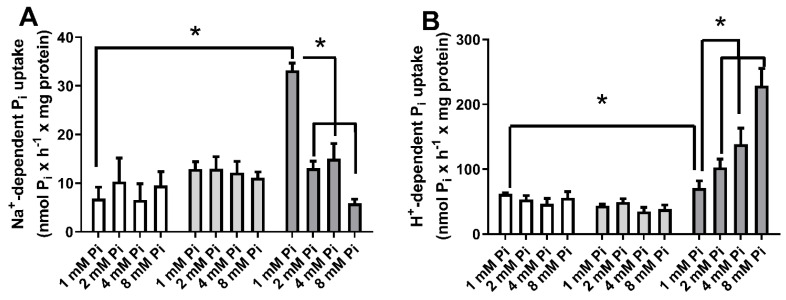
Effect of high inorganic phosphate concentrations on Na^+^-dependent or H^+^-dependent Pi uptake in breast cell lines. Different breast cell lines: MCF10-A (blank bars), MCF-7 (grey bars), and MDA-MB-231 (dark grey bars) were treated with various Pi concentrations (1, 2, 4 or 8 mM Pi) for 24 h. Na^+^-dependent (**A**) or H^+^-dependent (**B**) Pi uptake was measured for 1 h, as described in Section 4. Bars represent mean ± SE of at least three independent biological samples. * *p* < 0.05 indicates changes were significantly different, as assessed by ANOVA followed by Tukey’s multiple comparison test.

**Figure 2:**
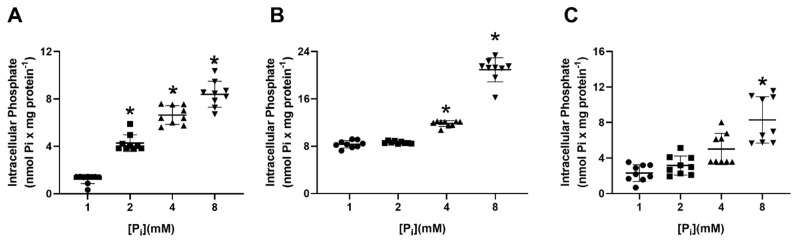
Effect of inorganic phosphate concentrations on total intracellular Pi in breast cell lines. Different breast cell lines: MCF10-A (blank bars) (**A**), MCF-7 (grey bars) (**B**), and MDA-MB-231 (dark grey bars) (**C**) were treated with various Pi concentrations (1, 2, 4 or 8 mM Pi) for 24 h and total intracellular Pi concentrations were determined, as described in Section 4. Bars represent mean ± SE of at least three independent biological samples. * *p* < 0.05 indicates changes were significantly different, as assessed by ANOVA followed by Tukey’s multiple comparison test.

Pi is absorbed by cells through Pi transporters [6]. The comparison of transcription levels of SLCs Pi transporters in MCF-7, MDA-MB-231, and MCF-10 cells, shows that Na^+^PiIIb is the principal transporter responsible for Na^+^:Pi uptake in all cells (Table S1 and Figure S1). In a previous study, our group demonstrated that MDA-MB-231 cells, after 24 h treatment with 2 mM Pi, showed inhibition of Na^+^-dependent Pi transport and, in a compensatory way, increased the H^+^-dependent Pi transport [33]. Therefore, to understand the effect of high Pi concentrations on breast cancer cell transporters, we measured Na^+^-dependent Pi transport (Figure 1A) and H^+^-dependent Pi transport (Figure 1B) in breast cell lines: a non-tumoral breast cell, MCF10-A; a luminal A breast cancer cell, MCF-7; and a TNBC cell, MDA-MB-231 in the presence of increasing concentrations of Pi (1, 2, 4 and 8 mM). As expected, treatment with high Pi inhibited Na^+^-dependent Pi transport and stimulated H^+^-dependent Pi transport. Nevertheless, this phenomenon was just seen in MDA-MB-231, with no effect on the other breast cell lines. In addition, an increase in total intracellular Pi concentration was observed in MCF-10A, MCF-7, and MDA-MB-231 cells after exposure to increasing extracellular Pi concentration for 24 h (Figure 2).

In this work, we used 1 mM Pi to represent the serum Pi concentration in healthy patients, and 2 mM and 4 mM Pi for serum Pi concentrations in cancer patients [8]. Seeking to understand how this effect occurs, we observed that 8 mM Pi was the concentration where we obtained the most pronounced regulatory effect. For a better analysis, in some experimental tests, we used the concentration 8 mM Pi as high Pi and 1 mM Pi as control.

### 2.2. Pi Stimulates Cell Migration and Adhesion Only in MDA-MB-231 without Impairing Viability

**Figure 3:**
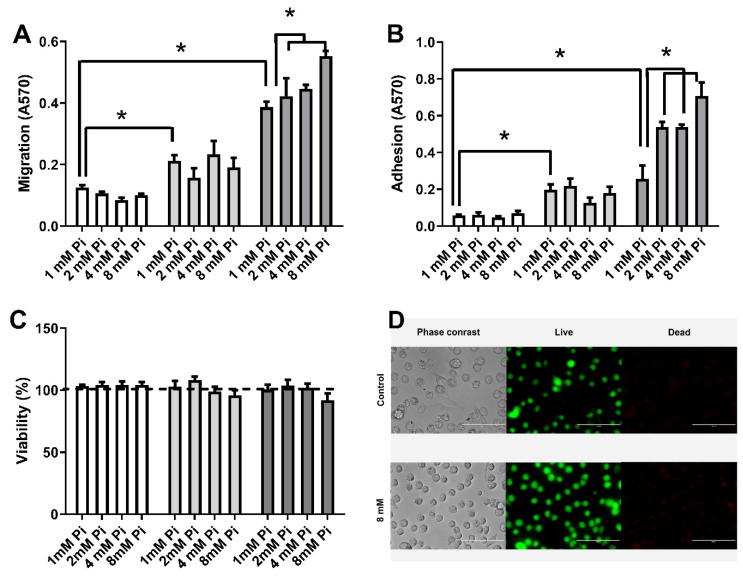
Effect of high Pi concentrations on the migration, adhesion to ECM and viability of the breast cell lines. Different breast cell lines: MCF10-A (white bars), MCF-7 (grey bars), and MDA-MB-231 (dark grey bars) were treated with various Pi concentrations (1, 2, 4 and 8 mM Pi) for 24 h. Migration (**A**), adhesion to ECM (**B**), and cell viability by MTS assay (**C**) were evaluated as described in Section 4. Bars represent mean ± SE of at least three independent biological samples. * *p* < 0.05 indicates changes significantly different, as assessed by ANOVA followed by Tukey’s multiple comparison test. Representative images of MDA-MB-231 cells treated with 1 mM (control) or 8 mM Pi for 24 h. Corresponding phase contrast images (left panels) and viability images (live/dead assay, green cells are alive and red cells are dead, right panels). Bars: 100 μm (**D**).

Lin et al. 2014, showed increased cell migration of MDA-MB-231 cells under high Pi concentrations (3 or 5 mM Pi) [13]. Using the same cell line, it was demonstrated that Na+-dependent Pi transporter and H^+^-dependent Pi transporter are related to migratory capacity [31,32]. Here, we evaluated migration and cell adhesion to extracellular matrix (ECM) at increasing Pi concentrations (1, 2, 4, or 8 mM Pi) in different breast cell lines. Only MDA-MB-231 cells had adhesion to ECM and cell migration stimulated by high Pi (Figure 3A,B). In these conditions, no changes could be seen in cell viability of any of the cell lines using MTS or live/dead assay (Figure 3C,D).

### 2.3. H_2_O_2_ as an Intracellular Messenger at Elevated Extracellular Pi in Breast Cells

**Figure 4:**
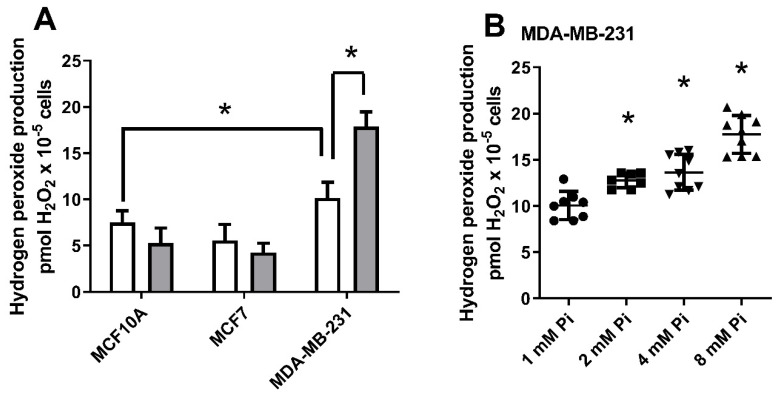
Influence of high Pi concentrations on hydrogen peroxide production by breast cell lines. H_2_O_2_ production was evaluated for 1 h in different breast cell lines: MCF10-A, MCF-7, and MDA-MB-231, as indicated on the abscissa, after 24 h treatment with basal Pi (1 mM Pi, white bars) or high Pi (8 mM Pi, grey bars) (**A**). MDA-MB-231 was treated at various Pi concentrations (1, 2, 4, and 8 mM Pi) for 24 h, and H_2_O_2_ production was evaluated at 1 h, as described in Section 4 (**B**). Bars represent mean ± SE of at least 3 independent biological samples. * *p* < 0.05 indicates changes significantly different, as assessed by ANOVA followed by Tukey’s multiple comparison test.

Currently, H_2_O_2_ is considered the main ROS involved in redox signalling processes, playing essential roles in the transduction pathways of many cancer cells [19,21]. As a pleiotropic signalling molecule, we aimed to investigate if H_2_O_2_ could also be involved in cellular events triggered by a high extracellular Pi concentration. Thus, H_2_O_2_ production was measured in different breast cell lines (MCF-10A, MCF-7, and MDA-MB-231) after 24 h of treatment with a high Pi concentration (8 mM). Compared to the other cell lines, MDA-MB-231 had the highest H_2_O_2_ production at 1 mM Pi concentration and was the only one to show a significant H_2_O_2_ stimulation at high Pi (2, 4, or 8 mM Pi) (Figure 4A,B).

**Figure 5:**
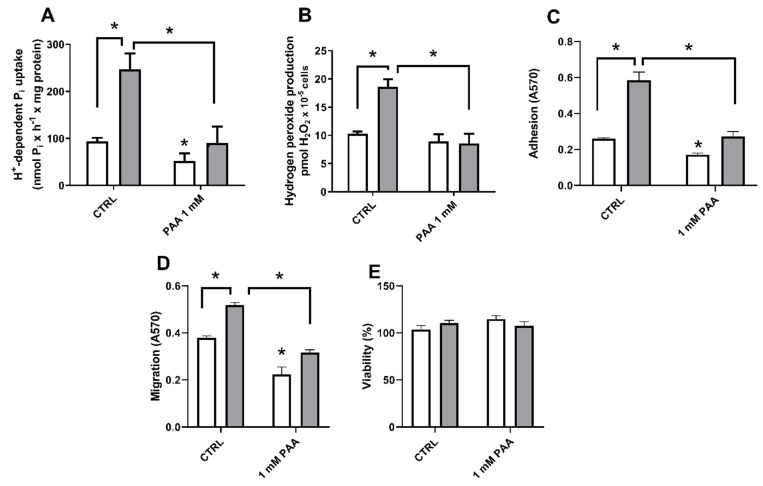
PAA effect on high Pi-induced Pi transport, migration and cell adhesion to ECM in MDA-MB-231 cells. MDA-MB-231 cells were treated with 1 mM Pi (blank bars) or 8 mM Pi (grey bars) for 24 h in the absence (control) or presence of an H^+^-dependent Pi transport inhibitor, PAA (1 mM). H^+^-dependent Pi uptake (**A**), H_2_O_2_ production (**B**), cell adhesion to ECM (**C**), cell migration (**D**), and cell viability (**E**) were performed as demonstrated in Section 4. Bars represent mean ± SE of at least 3 independent biological samples. * *p* < 0.05 indicates changes significantly different, as assessed by ANOVA followed by Tukey’s multiple comparison test.

Once high extracellular Pi stimulates only H^+^-dependent Pi transporter, we investigated if the addition of PAA (H^+^-dependent Pi transporter inhibitor) [32] could block Pi-induced H_2_O_2_ production accompanied by migration, adhesion to ECM, and cell viability. As expected, PAA inhibits H^+^-dependent Pi transport in cells treated with 1 or 8 mM Pi (Figure 5A), while in high Pi-induced conditions, the inhibition is more pronounced (63.5% inhibition, grey bars). The same profile is observed when PAA was tested on H_2_O_2_ production (Figure 5B), migration (Figure 5C), or cell adhesion to ECM (Figure 5D). PAA inhibition in high Pi-induced condition (8 mM Pi) leads to the same levels as in control conditions (1 mM Pi, without PAA). None of these treatments alters cell viability (Figure 5E).

### 2.4. The Source of H_2_O_2_ Production in Short Pi-Exposure

**Figure 6:**
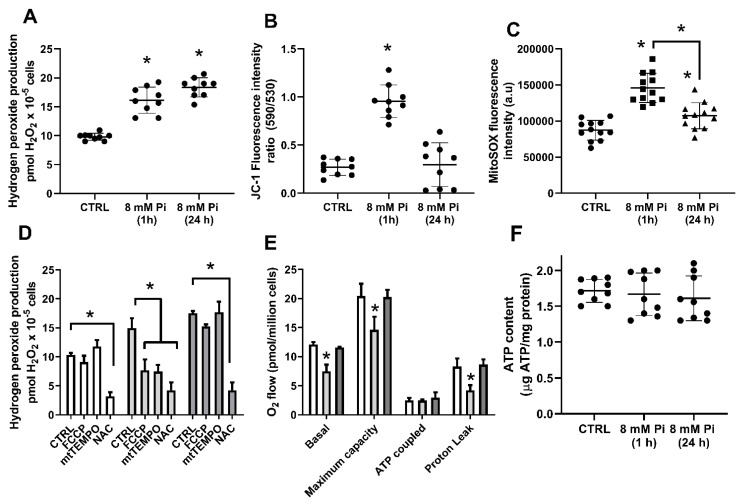
The H_2_O_2_ production source for short Pi-exposure in MDA-MB-231 cells. MDA-MB-231 cells were treated with 1 mM (CTRL) or 8 mM Pi for 1 h or 24 h. H_2_O_2_ production was evaluated for 1 h at 25 °C using Amplex Red (**A**), ΔΨm was measured with fluorescence dye JC-1 at room temperature (**B**) and mitochondrial ROS (superoxide generation) was measured using mitoSOX assay at 25 °C (**C**). MDA-MB-231 cells were treated with 1 mM Pi (white bars), 8 mM Pi for 1 h (light grey bars) or 8 mM Pi for 24 h (dark grey bars), H_2_O_2_ production was evaluated for 1 h in the presence of FCCP (1 µM), mitoTEMPO (100 nM) or NAC (5 mM) (**D**). MDA-MB-231 cells were treated with 1 mM Pi (white bars), 8 mM Pi for 1 h (light grey bars) or 8 mM Pi for 24 h (dark grey bars) and oxygen consumption was measured at basal levels, maximum capacity (1 µM FCCP), ATP coupled (basal levels of oxygen consumption minus values in the presence of 2.6 nM oligomycin) and proton leak (2.6 nM oligomycin) (**E**). ATP content was evaluated in MDA-MB-231 cells treated with 1 mM Pi (CTRL) or 8 mM Pi for 1 h and 24 h as indicated (**F**). Results represent mean ± SE of at least 3 independent biological samples. * *p* < 0.05 indicates changes significantly different, as assessed by ANOVA followed by Tukey’s multiple comparison test.

The mitochondrial electron transport chain (ETC) and the transmembrane NADPH oxidases can account for more than 50% of the total endogenous ROS production in a cell [19]. In insulin-releasing cells, elevated extracellular Pi (5 mM), after 30 or 60 min treatment, was shown to increase the mitochondrial membrane potential and O2^·−^ generation [26]. In this sense, we first evaluated if mitochondria would be the primary source of Pi-induced H_2_O_2_ production. Using MDA-MB-231 cells at different times after incubation with high Pi (8 mM), 1 h (short exposure) or 24 h (long exposure), we could see an increase in H_2_O_2_ levels up to 50% at 1 h treatment, while it almost doubled at 24 h treatment (Figure 6A). Mitochondria can produce ROS through the electron leak in the respiratory chain when its membrane is hyperpolarized (high ΔΨm). This condition results in lower electron transfer and partial reduction of oxygen to O2^·−^, which can then be reduced to H_2_O_2_ spontaneously or by the action of superoxide dismutase (SOD) [34].

MDA-MB-231 cells’ ΔΨm was measured after 1 h or 24 h treatment with 8 mM Pi through the use of a MitoProbe JC-1 assay. The results showed an increase in ΔΨm only after 1 h treatment. Interestingly, for 24 h treatment, no modulation was found (Figure 6B and Figure S2). To assess whether mitochondria was the source of high Pi-induced ROS, we used mitoSOX, a superoxide-sensitive fluorescence dye selective targeting to mitochondria, after 1 h or 24 h treatment with 8 mM Pi. Incubation with high Pi increased the mitochondrial superoxide generation (Figure 6C).

To confirm that mitochondrial membrane hyperpolarization was required for the observed Pi-induced H_2_O_2_ production after a short exposure, we pretreated cells with the protonophore FCCP, which collapses the electrochemical gradient, decreasing mitochondrial ROS generation. In addition, we have used the mitochondrial antioxidant mitoTEMPO to specifically scavenger mitochondrial Pi-induced ROS production. Figure 6D shows that the Pi-induced H_2_O_2_ production under short exposure was abolished in the presence of FCCP (1 μM) and mitoTEMPO (100 nM). Nevertheless, FCCP and mitoTEMPO did not modulate H_2_O_2_ production under long exposure conditions. As a positive control, we used the ROS scavenger N-acetyl-cysteine (NAC), which showed a significant reduction of H_2_O_2_ production after 1 h and 24 h treatment at high Pi (Figure 6D). After that, using the same conditions, we analysed oxygen consumption. Figure 6E shows a decrease in oxygen consumption only under 1 h treatment. However, no modulation was observed in ATP content when cells were treated with 8 mM Pi for 1 h or 24 h (Figure 6F). Taken together, the results indicate that mitochondrial ROS generation may occur under short Pi-exposure, but not under long Pi-exposure.

### 2.5. The H_2_O_2_ Production in Long Pi-Exposure

**Figure 7:**
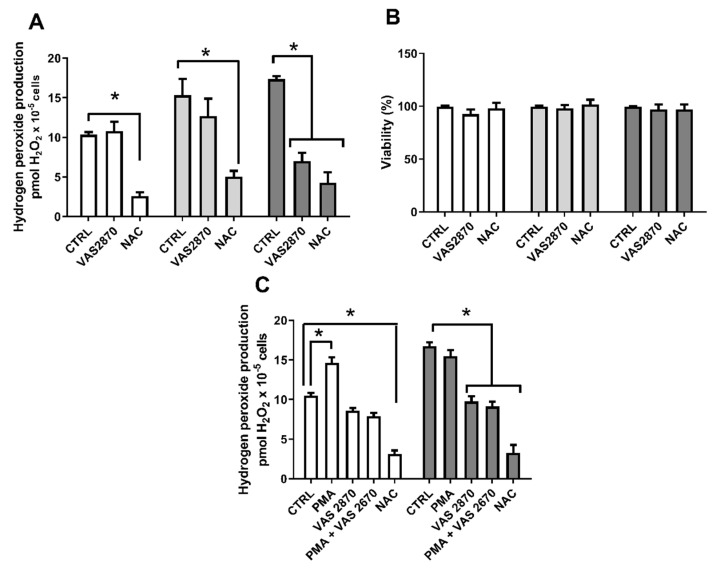
The H_2_O_2_ production source for long Pi-exposure in MDA-MB-231 cells. MDA-MB-231 cells were treated with 1 mM Pi (white bars), 8 mM Pi for 1 h (light grey bars) or 24 h (dark grey bars). Then, H_2_O_2_ production was evaluated for 1 h using Amplex Red in the presence of 10 µM VAS2870 and 5 mM NAC (**A**). Cell viability (**B**) was evaluated as described in Section 4. H_2_O_2_ production was evaluated in the presence of 1 nM PMA, 10 µM VAS2870, 1 nM PMA plus 10 µM VAS2870 and 5 mM NAC (5 mM) (**C**). Bars represent mean ± SE of at least 3 independent biological samples. * *p* < 0.05 indicates changes significantly different, as assessed by ANOVA followed by Tukey’s multiple comparison test.

Since mitochondria seem not to be responsible for ROS generation under long Pi-exposure, we hypothesized a possible involvement of NOX enzymes [34,35,36,37,38]. The triazol pyrimidine derivative, VAS2870 (3-benzyl-7-(2-benzoxazolyl)thio-1,2,3-triazolo [4,5-d]pyrimidine) is characterized as a NOX inhibitor, without antioxidant properties or attenuation of xanthine oxidase activity [37]. The results show that 10 μM VAS2870 was not able to affect Pi-induced H_2_O_2_ production after 1 h treatment. Nonetheless, VAS2870 abrogated H_2_O_2_ generation under chronic treatment with 8 mM Pi without affecting cell viability, raising the possibility that the secondary source of Pi-induced H_2_O_2_ production could be a NOX (Figure 7A,B).

Pi overload can induce ROS production via NADPH oxidase by activating conventional PKC in endothelial cells [29]. Here, we have evaluated PKC participation in Pi-induced H_2_O_2_ production. Thus, the PKC stimulator, phorbol 12-myristate 13-acetate (PMA), is able to increase the H_2_O_2_ production in control conditions (Figure 7C). The high production of H_2_O_2_ induced by 8 mM Pi treatment for 24 h reached the same levels as in PMA-treated control cells (1 mM Pi), whereas no increment was seen when cells were treated with PMA and 8 mM Pi together for 24 h (Figure 6). The NOX inhibitor (VAS 2870) was able to block the generation of H_2_O_2_ induced by high Pi, as well as by PMA (Figure 7C). The results suggest the participation of a NADPH oxidase-activated by PKC in this chronic high Pi condition.

**Figure 8:**
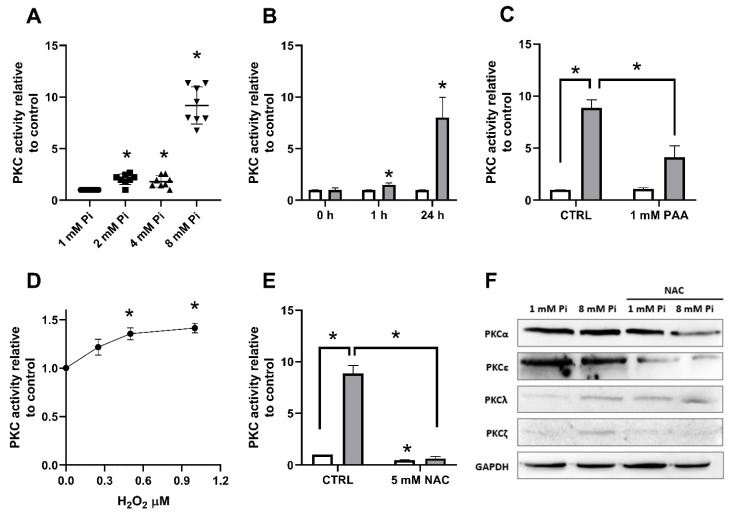
PKC is involved in Pi-induced H_2_O_2_ production in MDA-MB-231 cells. PKC activity was measured, as described in Section 4, in MDA-MB-231 cells treated with various Pi concentrations (1, 2, 4, or 8 mM Pi) for 24 h (**A**); or with 1 mM (white bars) or 8 mM Pi (grey bars) for 1 h or 24 h (**B**). The same conditions were evaluated (1 mM Pi—white bars, 8 mM Pi—grey bars) in the absence (control) or presence of PAA (1 mM) for 24 h (**C**). Increasing H_2_O_2_ concentrations (0–1 μM) were added in the reaction mixture and PKC activity was analyzed for 10 min (**D**). MDA-MB-231 cells were treated with 1 mM (blank bars) or 8 mM Pi (grey bars) in the absence (control) or presence of NAC (5 mM) for PKC activity evaluation (**E**). Representative immunodetections of PKC isoforms: PKCα, PKCε, PKCλ, and PKCζ, in cells maintained at 1 mM or 8 mM Pi, in the presence or absence of NAC for 24 h. GAPDH was used as loading control (**F**). Bars represent mean ± SE of at least 3 independent biological samples. * *p* < 0.05 indicates changes significantly different, as assessed by ANOVA followed by Tukey’s multiple comparison test.

To investigate if PKC would be activated by Pi-induced H_2_O_2_ production, we have measured PKC activity in the presence of increasing Pi concentrations (1, 2 or 8 mM Pi) for 24 h treatment. Figure 8A shows that PKC activity is similarly stimulated by 2 or 4 mM Pi, 0.8 fold in relation to the control cells. Notwithstanding, it was 8-fold stimulated higher in high Pi (8 mM). This phenomenon was also true for 1 h treatment, albeit less prominent than in 24 h (Figure 8B). Interesting, H^+^-dependent Pi transport inhibitor PAA suppresses PKC activity stimulation by high Pi at 24 h (Figure 8C). To test whether H_2_O_2_ was required for PKC activation, increasing H_2_O_2_ concentrations was added to the reaction mixture. Figure 8D shows that PKC activity is stimulated by increasing H_2_O_2_ concentrations, and this effect is abrogated by NAC (Figure 8E). The expression of different PKC isoforms (αPKC, the conventional PKC isoform; εPKC, the novel PKC isoform, ζPKC and λPKC, atypical PKCs isoforms) were analyzed in cells maintained at 1 mM or 8 mM Pi, in the presence or absence of NAC for 24 h. An increased expression of atypical PKCs isoforms (ζPKC and λPKC) was observed at 8 mM Pi (Figure 8F). Furthermore, a reduction for most PKC isoforms tested (αPKC, εPKC and ζPKC) was notified at high Pi in the presence of NAC (Figure 8F).

### 2.6. Pi-Generated H_2_O_2_ Production Modulates Pi Transporters, Migration, and Cell Adhesion to ECM

**Figure 9:**
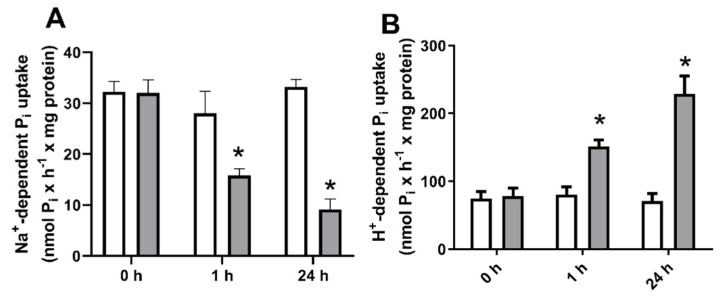
Effect of high Pi Na^+^-dependent or H^+^-dependent Pi transport for short (1 h) or long (24 h) Pi-exposure of MDA-MB-231 cells. MDA-MB-231 cells were treated with 1 (white bars) or 8 mM Pi (grey bars) for different times of exposure (0, 1 and 24 h). Na^+^-dependent (**A**) or H^+^-dependent (**B**) Pi uptake was done for 1 h, as described in Section 4. The results are mean ± SE of at least three independent biological samples. * *p* < 0.05 indicates changes significantly different, as assessed by ANOVA followed by Tukey’s multiple comparison test.

Next, we investigated whether high Pi regulates Pi transporters through H_2_O_2_ generation. The effect on Pi transporters of short and long exposure to high Pi (8 mM) was evaluated. Na^+^-dependent Pi transport showed inhibition in both periods of Pi exposure (Figure 9A). The opposite was observed in H^+^-dependent Pi transport with significant stimulation in response to either 1 h or 24 h of treatment with high Pi (Figure 9B).

**Figure 10:**
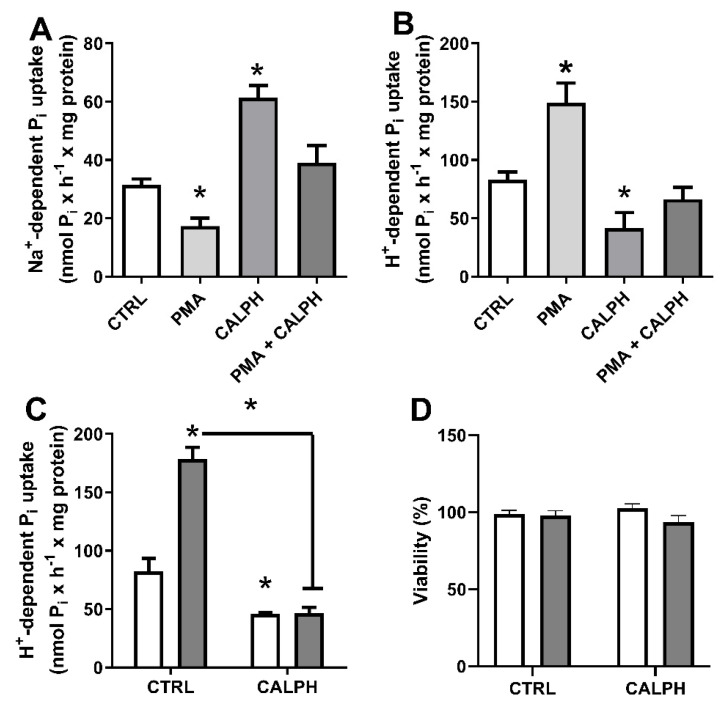
Role of PKC in modulation of Pi uptake. Na^+^-dependent (**A**) or H^+^-dependent (**B**) Pi uptake were performed for 1 h in the presence of PKC stimulator PMA (1 nM), PKC inhibitor Calphostin C (CALPH:50 nM) or PMA plus CALPH as indicated. MDA-MB-231 cells were treated with 1 mM Pi (white bars) or 8 mM Pi (grey bars) in the absence (control) or presence of CALPH (50 nM) for 24 h and H^+^-dependent Pi uptake (**C**) and cell viability (**D**) were performed as indicated in Section 4. The results are mean ± SE of at least 3 independent biological samples. * *p* < 0.05 indicates changes significantly different, as assessed by ANOVA followed by Tukey’s multiple comparison test.

As PKC is activated by H_2_O_2_, we sought to evaluate the effect of PKC on the modulation of Pi transporters. Na^+^-dependent or H^+^-dependent Pi transport levels were measured in the presence of PMA (PKC stimulator), calphostin C (PKC inhibitor), or PMA plus calphostin C (Figure 10). PMA inhibited, whereas calphostin C stimulated the Na^+^-dependent Pi transport and both modulators had the opposite effect on H^+^-dependent Pi transporter. In both transporters, calphostin C was able to inhibit PMA effect (Figure 10A,B). In addition, the role of PKC in Pi-induced H_2_O_2_ production was also evaluated for Pi transport activity. The increased H^+^-dependent Pi transport at high Pi by long exposure (24 h treatment) was blocked in the presence of the PKC inhibitor (Figure 10C) without affecting cell viability (Figure 10D). The results suggest a modulation of Pi transporters (at least H^+^-dependent) by Pi-induced PKC activity.

**Figure 11:**
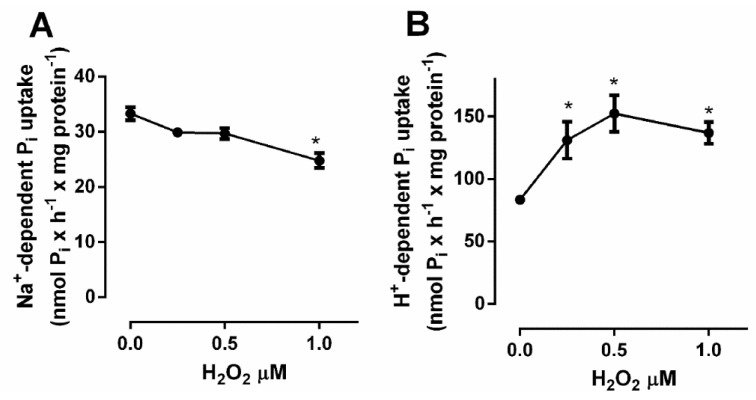
Effect of H_2_O_2_ concentrations on Na^+^-dependent or H^+^-dependent Pi transport in MDA-MB-231 cells. Na^+^-dependent (**A**) or H^+^-dependent (**B**) Pi uptake was done for 1 h in the presence of increased H_2_O_2_ (0–1 µM) was added to the reaction mixture, as described in Section 4. The results are mean ± SE of at least three independent biological samples. * *p* < 0.05 indicates changes significantly different, as assessed by ANOVA followed by Tukey’s multiple comparison test.

Furthermore, the Pi transport was assayed in the presence of increasing concentrations of H_2_O_2_ (0–1 μM). Figure 11A shows that H_2_O_2_ was able to inhibit the Na^+^-dependent Pi transporter in a dose-dependent fashion, at the same time that it stimulated the H^+^-dependent Pi transporter (Figure 11B).

**Figure 12:**
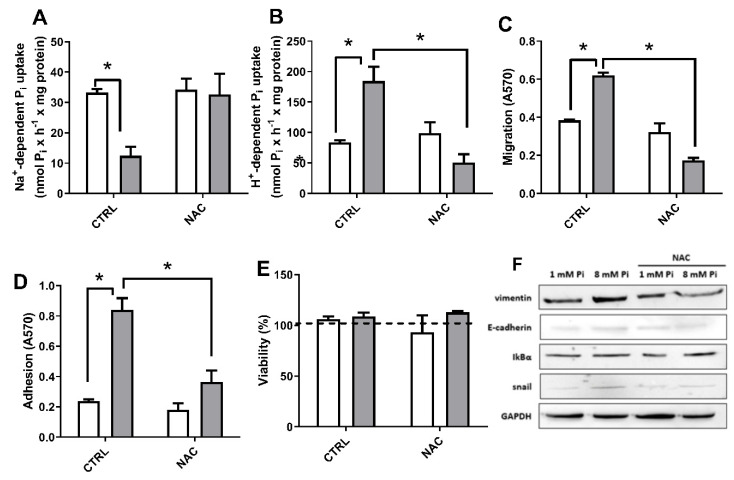
NAC effect on high Pi-induced Pi transport, migration, and cell adhesion to ECM in MDA-MB-231 cells. MDA-MB-231 cells were treated with 1 mM Pi (white bars) or 8 mM Pi (grey bars) in the absence (control) or presence of NAC (5 mM) for 24 h. Na^+^-dependent (**A**) or H^+^-dependent (**B**) Pi uptake was done for 1 h. Migration (**C**), adhesion to ECM (**D**), and cell viability (**E**) were performed as described in Section 4. Bars represent mean ± SE of at least three independent biological samples. * *p* < 0.05 indicates changes significantly different, as assessed by ANOVA followed by Tukey’s multiple comparison test. Representative immunodetections of, vimentin, E-cadherin, snail and IkBα in cells maintained at 1- or 8-mM Pi, in the presence or absence of NAC (**F**). GAPDH was used as loading control.

To investigate whether Pi-generated H_2_O_2_ production can regulate Pi transporters and tumoral processes in MDA-MB-231 cells, we used NAC (5 mM) at high Pi concentrations (8 mM Pi) within a 24-h treatment. The inhibition of Na^+^-dependent Pi transport caused by high Pi was reversed in the presence of NAC (Figure 12A) and as expected, the high Pi-induced stimulation of H^+^-dependent Pi transport was also reversed (Figure 12B). Stimulation of migration and cell adhesion to ECM by high Pi was also inhibited by NAC (Figure 12C,D), while cell viability was not affected (Figure 12E).

We also analysed the marker proteins (E-cadherin and vimentin) and signalling pathways intermediates (IkBα or Snail), associated with migration capacity and EMT, by immunodetections (Figure 12F). MDA-MB-231 cells were grown for 24 h in 1 mM or 8 mM Pi, in the presence or absence of NAC. While control cells presented low expression of E-cadherin and high expression of vimentin (Figure 12F), cells grown in the presence of 8 mM Pi for 24 h presented induced expression of vimentin and snail, the critical transcription factor in EMT (Figure 12F). In addition, the high Pi-induced snail and vimentin expression was blocked in the presence of NAC. Furthermore, the immunodetection for IkBα (NF-κB inhibitor-protein) was slightly increased by 8 mM Pi with NAC, which may infer a low NF-κB activity. These observations strongly suggest that EMT is stimulated by Pi-induced ROS production and possibly regulated by NF-κB-dependent activation of Snail.

## 3. Discussion

Papaloucas et al. (2014) showed that cancer patients have higher serum Pi (2.52 ± 0.72 mmol/L) compared with healthy individuals (1.09 ± 0.19 mmol/L) [8]. According to the growth-rate hypothesis, high Pi serum would be necessary to support the increased growth rates and tumour progression [7]. It was recently hypothesized by Brown (2019) that during inflammatory hyperaemia, blood flow rate and blood volume together with hyperphosphatemia (a serum phosphate concentration higher than 1.45 mM) promotes tumorigenesis in accordance with the GRH hypothesis [9]. In lung cancer, a high Pi intake (a diet fortified with 1.0% Pi) led to pulmonary tumour progression via the Akt-mTOR regulatory pathway [10]. In skin cancer, a diet fortified with 1.2% Pi in mice showed promotion of tumorigenesis through activation of a signalling pathway consisting of ras and ERK1/2 [12]. Therefore, we considered 1 mM Pi as the physiological Pi concentration and above this limit, as high Pi (2, 4, and 8 mM Pi). A higher concentration of Pi (8 mM), although beyond the regular serum Pi range in cancer patients, showed a greater regulation of Pi transporters and H_2_O_2_ production. For this reason, it was chosen in some tests to give a more pronounced effect in the experimental analysis.

**Figure 13:**
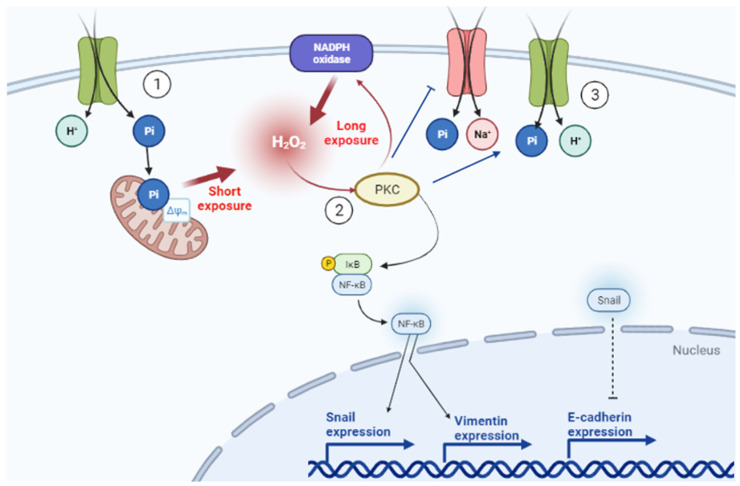
Schematic representation of Pi-induced peroxide hydrogen production and its role in Pi transporters modulation in MDA-MB-231 cells. (**1**) An increase in intracellular Pi occurs through specific Pi transport sensitive to PAA. In a short Pi exposure, Pi hyperpolarizes the mitochondrial membrane and increases the release of mitochondrial H_2_O_2_ sensitive to FCCP and mitoTEMPO. In long Pi exposure, nicotinamide adenine dinucleotide phosphate (NADPH) oxidase (NOX), sensitive to VAS2870, is the secondary source of H_2_O_2_ production. (**2**) Increase of intracellular H_2_O_2_ activates PKC, which possibly phosphorylate and activate IKK, a kinase of IkB, the inhibitor that hijacks the transcription factor NF-kB in cytoplasm, which once phosphorylated releases NF-kB to enter in the nucleus to start the transcription of genes related to Snail (an E-cadherin repressor expression), vimentin and E-cadherin. Moreover, (**3**) PKC activated by H_2_O_2_ inhibits Na^+^-dependent Pi transporter, stimulates H^+^-dependent Pi transporter, and therefore, stimulates migration and cell adhesion to ECM. Figure created with BioRender.com.

It has been demonstrated by many studies that Pi-mediated ROS production occurs in different cell types: rat osteosarcoma (MR-106 cells), insulin-secreting cells (INS-IE), human endothelial cells (EAhy926) and BAECs [22,23,24,25,26,27,28]. Here, we show for the first time that Pi-mediates ROS production in triple-negative breast cancer cells (MDA-MB-231) (Figure 4). Although previous works have thought that Pi-induced H_2_O_2_ production was mitochondrial or to have a NOXs source, in this work, we suggest the participation of H_2_O_2_ as a signalling molecule to regulate the activities of both Pi transporters, Na^+^-dependent and H^+^-dependent, in MDA-MB-231 cells exposed to high extracellular Pi (Figure 13). In a short exposure time (1 h), Pi hyperpolarizes the mitochondrial inner membrane, increases the release of mitochondrial H_2_O_2_, and inhibits O_2_ consumption [24,27] (Figure 6). The driving force of the mitochondrial proton is the sum of the electrical potential (ΔΨm) and the chemical potential determined by ΔpH across the inner mitochondrial membrane [24,27]. The Pi co-transporter possibly mediates the hyperpolarization with H^+^ (or in exchange for OH), which decreases ΔpH and increases the ΔΨm [24,25]. The increase of ΔΨm promotes electron leak into the ETC and consequently superoxide production and H_2_O_2_ by spontaneously reduction or action of SOD [34,36]. As no difference in O_2_ consumption coupled to ATP and ATP content was observed (Figure 6D,E), we believe that this effect has a role in cell signalling, mainly due to the diffusibility of H_2_O_2_ across the plasma membrane.

We believe that the primary source of H_2_O_2_ production after a short exposure is via mitochondria for the following reasons: (1) The hyperpolarization of the electrical potential of the mitochondrial membrane only occurred for 1 h and not 24 h (Figure 6B), because this effect is rapid and reversible [24,25,26,27]; (2) Pi-induced H_2_O_2_ production for 1 h was blocked by FCCP, which collapses the established electrochemical gradient, which was not possible to observe for 24 h (Figure 6C). (3) The mitochondrial ROS generation induced by high Pi was prominent just for 1 h treatment, as mitoTEMPO antioxidant was most effective at this same condition. (4) Oxygen consumption was only impaired in a short Pi exposure, but not during prolonged exposure (Figure 6E), showing that ETC functionality was possibly affected by Pi-mediated hyperpolarization. This is in agreement with previous reports showing that elevated ΔΨm favours electron transport chain-dependent ROS synthesis [23,24]. All of these reasons also led to the conviction that the H_2_O_2_ production by long Pi-exposure is not from a mitochondrial source.

Many functions of mitochondria are closely linked to their morphology, and there is a delicate balance between the events of fusion and fission. In cancer cells, this balance is shifted, mainly to fission by tumour factors that act on the expression of mitochondrial genes responsible for mitochondrial maintenance, negatively impacting the transfer of electrons in the CTE, promoting ROS leakage [39,40]. It was recently demonstrated by Sarmiento-Salinas et al. [40] that there were different effects of ROS production in different breast cell lines: MCF10-A and MCF-7 (complete tubular mitochondrial morphology), MDA-MB-231 (partial mitochondrial fragmentation) and MDA-MB-468 (complete mitochondrial fragmentation) [40]. The total ROS production was higher in the MDA-MB-231 and MDA-MB-468 cells compared to the MCF-7 and MCF10-A, partially following what we have shown in Figure 4A. However, the production of mitochondrial ROS was only elevated in the MDA-MB-468 cells, possibly due to the total fragmentation of the mitochondrial membrane [40]. Here, among the cell lines tested (MCF10-A, MCF-7, and MDA-MB-231), the H_2_O_2_ production induced by Pi was unique in the MDA-MB-231 line (Figure 4A), possibly due to the mitochondrial morphology being a pre-disposition factor to an increased H_2_O_2_ release when stimulated by Pi. However, these observations need to be extended to other breast cancer cell lines.

Furthermore, other mitochondrial effects can be modulated by Pi-induced ROS production not evaluated in this work. For example, it has been demonstrated that Pi stimulates lipid peroxidation in mitochondria isolated from rat livers [41]. It was also demonstrated that Pi-induced ROS production activated the opening of the mitochondrial permeability transition (PT) pore in Rat insulinoma INS-1E cells [22,23,24,25,26].

In MDA-MB-231 cells, Sarmiento-Salinas et al. [40] showed that 50% of total ROS production is via mitochondria, and other sources of ROS contribute to the total ROS production to support oncogenic signalling [40]. In addition to CTE, another primary source of endogenous ROS is NADPH oxidases. The NOX family consists of seven isoforms, NOX1–5 and DUOX1–2. In breast epithelial cells (MCF10-A), reduced expression of NOX4, DUOX1, and DUOX2 has been demonstrated [35,36,42]. On the other hand, in MDA-MB-231 cells, there is moderate expression of NOX2 and NOX3, slightly higher of NOX5, and high expression of NOX4 [42].

Shuto et al. [29] showed that the overload of Pi in bovine aortic endothelial cells promoted an increase in the production of ROS via a NADPH oxidase by observing the decreased production of ROS in the presence of NOX inhibitor [29]. Here, we have used a specific NOX inhibitor, VAS2870 [36], and this compound was only able to inhibit the production of H_2_O_2_ in a long Pi exposure time (not in short exposure) in MDA-MB-231 cells (Figure 6), suggesting NOX as the primary source of H_2_O_2_ in long exposures (24 h) to high Pi (8 mM Pi) (Figure 13). Nevertheless, more studies are needed to prove the participation of NOX.

Because NOX-mediated H_2_O_2_ production stimulated by Pi is time-dependent (only in long exposure), possibly another H_2_O_2_ or ROS source could have a co-relation with the NOX activation. It has been extensively studied that NOXs can be regulated through phosphorylation of different regulatory subunits (p22^phox^, p40^phox^, p47^phox^, and p67^phox^) by different cell signalling pathways, including MAPK, PI3K, PKA and PKC [35,36]. Shuto et al. [29] also demonstrated that Pi overload could induce ROS production via NADPH oxidase by activating conventional PKC [29]. It is known the presence of two pairs of zinc fingers within the regulatory domain in the PKC structure. H_2_O_2_ and other oxidants can destroy the zinc finger conformation, and the autoinhibition is relieved, resulting in a PKC form that is catalytically active in the absence of Ca^2+^ or phospholipids [43]. Here, we suggest that PKC activated by H_2_O_2_ can increase H_2_O_2_ production via NOX activation in high Pi treatment for 24 h (Figure 8).

Cancer cells need to expend energy to adhere and migrate effectively through the complex architecture of the extracellular matrix, usually through the dephosphorylation of ATP to ADP, when an actin monomer binds to an actin filament performing the filament polarization [44,45]. Recently, we showed that the MDA-MB-231 cell has a higher level of Pi transport, either Na^+^-dependent or H^+^-dependent, compared to other breast cancer cells. Additionally, high extracellular Pi (3 and 5 mM) acts as a migratory stimulator in lung (A549) and breast cancer cells (MDA-MB-231) [13]. Therefore, it is believed that the tumour cell requires more Pi to supply the energy needs of migration and cell adhesion to ECM. Indeed, the Pi transport plays a fundamental role in stimulating migration and adhesion in high extracellular Pi [6,31,32,33].

Pi enters the cells via Pi transporters either by Na^+^-dependent or H^+^-dependent Pi transporters. Recently, two Pi transporters with a different mechanism and substrate affinity were characterized: the Na^+^-dependent Pi transport has a high affinity for Pi (transports more in low concentrations, K_m_ = 0.08 mM Pi), and the H^+^-dependent Pi transport has a low affinity for Pi (transports more in high concentrations, K_m_ = 1.4 mM Pi) [31,32]. A decreased Na^+^-dependent Pi transport was observed at high extracellular Pi concentrations (2 and 8 mM), and, as a compensatory mechanism, H^+^-dependent Pi transport was stimulated (Figure 1) [32]. Considering kinetic behaviour, the H^+^-dependent transporter would exert a more significant contribution to the intracellular pool of Pi under high concentrations of extracellular Pi (Figure 2 and Figure 13) [32]. A similar effect was observed in the biochemical characterization of Pi transport in intestinal cells, Caco2BBE, when they were grown in 1 mM Pi (control condition) and 4 mM Pi, which mimics the high concentration of Pi in the intestinal lumen. Although all conditions presented a Na^+^-independent Pi uptake, Caco2BBE growth in high Pi (4 mM) had an increased transport of Na^+^-independent Pi transport with a Km value of 0.155 ± 0.025 mM Pi compared to control conditions (1 mM) with a K_m_ value of 0.071 ± 0.020 mM Pi [46]. In addition, we recently showed PAA as an inhibitor of the H^+^-dependent Pi transporter [32]. In this work, PAA was able to block the stimulation of the H^+^-dependent Pi transporter and consequently the H_2_O_2_ production, migration, cell adhesion to ECM, and PKC activity induced by Pi (Figure 5, Figure 8C and Figure 13).

Because of Pi’s ability to induce H_2_O_2_ production, whether at 1 h or 24 h, we also showed that high Pi inhibits the Na^+^-dependent Pi transport and stimulates the H^+^-dependent Pi transport (Figure 6). We observed the same modulation profile Pi-induced by H_2_O_2_ added in control conditions to the Na^+^-dependent or H^+^-dependent Pi transport (Figure 7), which were reversed by a ROS scavenger, NAC (Figure 12A,B). An explanation for the H_2_O_2_-Pi transporters regulation would be a direct sensitivity to H_2_O_2_ (Figure 12B). Furthermore, other signalling pathways regulated by H_2_O_2_ could act as an intermediate regulator for Pi transporters. Most cell signaling exerted by H_2_O_2_ will occur in a limited range of low concentration, otherwise, as a very diffusible oxidant molecule, it would lead to oxidative stress at high concentrations. Work with such low concentrations of H_2_O_2_ is an additional challenge due to its instability in exogenous media. Nevertheless, addition of exogenous low concentrations of H_2_O_2_ was able to increase PKC activity and modulate Pi transporters. This is in agreement with the existing steep gradient of H_2_O_2_ concentration (100–500-fold) between extracellular and intracellular spaces, which can reach an estimated range of 1–10 nM in the intracellular medium [19].

Cancer cells that exhibit persistent metabolic oxidative stress compared to healthy cells generally contain a mixture of DNA lesions and activation of oncogenes, promoting a change in the expression of several proteins [20]. In addition, H_2_O_2_ reversibly oxidizes the cysteine thiol phosphatase groups, such as phosphatase and tensin homologue (PTEN), protein tyrosine phosphatase 1B (PTP1B) and protein phosphatase 2, which cause loss of activity and consequently promote the activation of the proliferation pathway PI3K/Akt/mTOR [47]. Some studies have reported that Na^+^-dependent Pi transport can be regulated by this pathway. In the brush border membrane of the kidney of rats, parathyroid hormone (PTH) and a high Pi-diet promote internalization and subsequent lysosomal routing of the type II Na/Pi co-transporter via PKC activation [48], which would be involved in this endocytic regulatory cascade (PTH1R-PKC-NHERF-1 way) [49,50]. Because of few studies about H^+^-dependent Pi transport, no regulatory signalling pathway has been demonstrated. However, only one Pi (H^+^-dependent) transporter would possibly be regulated by H_2_O_2_ and consequently would intrinsically regulate another Pi (Na^+^-dependent) transporter. Bowen and Levison [51] showed in Ehrlich ascites tumour cells that H^+^ stimulation of Na^+^-independent Pi transport inhibits Na^+^-dependent Pi transport, possibly by the interaction of intracellular H^+^ with an intracellular site that regulates the Na^+^-dependent Pi transporter [51].

As shown, H_2_O_2_ can activate PKC, and Pi-induced H_2_O_2_ production affects PKC activity. Figure 10 shows the modulation of Pi transporters by high extracellular Pi through PKC activation. When PMA activates PKC, Na^+^-dependent Pi transport is decreased, and H^+^-dependent is increased. The opposite is observed when PKC is inhibited by calphostin C (Figure 10). However, Figure 6 shows that PMA modulates H_2_O_2_ production, which could be related to the activation of NOX since this enzyme can be phosphorylated by PKC (Figure 13). Further experiments are needed to elucidate the precise modulation of PKC in Pi transporters (Figure 10). Analysis of the primary sequence of NaPiIIb, the principal transporter responsible for Na^+^:Pi uptake (Figure S1), shows at least 16 predicted phosphorylation sites specific for PKC, where presumably a direct activation could occur (Table S2). In addition, the increased H^+^-dependent Pi transport at high Pi by long exposure (24 h) was blocked in the presence of the PKC inhibitor showing a possible mechanism of regulation of Pi transporters when high Pi stimulates the production of H_2_O_2_. Regarding decreased Na^+^-dependent Pi transport, the decreased expression of Na^+^-dependent Pi transport in high Pi (2 mM), as previously reported [32], makes it challenging to perform experimental assays related to PKC regulation in Na^+^-dependent Pi transport.

In this work, we showed a unique Na^+^-dependent inhibition and H^+^-dependent Pi transport stimulus by high Pi (24 h) in the triple-negative breast cancer cell line, MDA-MB-231 (Figure 1) when compared to other breast cell lines: MCF-7 (Luminal A) or MCF10-A (non-tumoral breast cell line). Although the level of total Pi was increased in all cell lines (MCF-10A, MCF-7 and MDA-MB-231) in response to elevated extracellular Pi, cell migration and adhesion were stimulated only in MDA-MB-231 cells in response to an increase in extracellular Pi (Figure 2 and Figure 3). The measured total intracellular Pi concentration is not proportional to the total amount of Pi uptake by Pi transporters, since the Pi would be complexed with biomolecules for different locations, including extracellular vesicles (EV), which has already been shown to have a correlation with high invasive capacity of MDA-MB-231 cells [52]. Moreover, only MDA-MB-231 cells showed significant H_2_O_2_ production stimulation in response to high Pi (2 folds higher, Figure 4A). Even though was observed a high modulation on Na^+^-dependent Pi transport (7 folds lower) and H^+^-dependent Pi transport (3 folds higher) in response to high Pi (Figure 1), the addition of 1 μM H_2_O_2_ only stimulated H^+^-dependent Pi transport by 30% as well inhibits Na^+^-dependent Pi transport by 30% (Figure 11).

Triple-negative breast cancer cells have higher ROS production when compared to luminal or non-tumoral breast cells [40]. Studies have shown that elevated levels of H_2_O_2_ generated by metastatic breast adenocarcinoma cells promote the regulation of epithelium-mesenchymal transition markers [53,54]. In the short term, we showed a Pi-induced H_2_O_2_ generation by mitochondria. However, in this work, we chose to focus on prolonged exposure to Pi for metastatic processes as it comprises the total H_2_O_2_ generation induced by high Pi concentration. We demonstrated that Pi stimulated cell migration, cell adhesion to ECM, and induced EMT (Figure 3 and Figure 12). Additionally, the antioxidant NAC prevented the Pi-induced metastatic process and vimentin expression (Figure 12). As expected, no modulation of E-cadherin levels was observed at 8 mM Pi, and with NAC, once in all conditions, relative immunostaining of Snail (a potent transcriptional repressor of E-cadherin) was observed. Therefore, we believe that Pi-induced H_2_O_2_ production can contribute to EMT and a higher metastatic capacity induced by Pi (Figure 13).

NF-κB is an essential transcription factor for EMT, inducing transcription factors such as Snail and mesenchymal markers, as vimentin [53,55]. Besides, the NF-κB is involved in ROS-induced EMT via Snail induction in mammary epithelial cells [54]. Here, we detect by Western blotting the IκBα, known as an NF-κB hijacker, by masking their NF-κB nuclear localization sequence, and thereby retaining it into the cytoplasm and preventing DNA binding [55]. Accordingly, higher cytoplasmic detection of IκBα in MDA-MB-231 cells at 8 mM Pi with NAC infers lower NF-κB activity, suggesting that NF-κB induces Snail and vimentin transcription, therefore participating on Pi-induced EMT and migration mediated by ROS released (Figure 13). Besides, PKC selectively regulates the activation of the NF-κB in response to oxidative stress [43]. In this work, we show reduced activity and expression of different PKC isoforms (PKCα, PKCε and PKCζ) at 8 mM Pi with NAC for 24 h. Atypical PKC Isoforms, as PKCζ, have a role in the EMT that characterizes the invasive phenotype associated with metastatic carcinomas [56]. We believe that PKC activity could be involved in the activation of NF-κB activity in Pi-induced EMT and cell migration mediated by ROS releasing since PKC is activated by ROS [43].

In summary, TNBC represents a challenge for medical clinics, either because they present higher metastatic aggressiveness compared to other breast cancer types or because they have few therapeutic targets [2,3]. The high concentration of serum Pi in patients with breast cancer can aggravate the condition because of the GRH or stimulation of the metastatic process [7,10]. Here, we showed that a high extracellular Pi induces the production of H_2_O_2_, whether in short or long exposure (Figure 13). In the short exposure, Pi hyperpolarizes the mitochondrial membrane, impairing the functioning of the ETC, with increased release of H_2_O_2_. After prolonged exposure to Pi, a source of H_2_O_2_ inhibited by VAS-2870, possibly a NADPH oxidase, would contribute to the production of H_2_O_2_. In addition, the TNBC subtype has an enhanced metastatic capacity, probably due to the high concentration of Pi, and the Pi-induced H_2_O_2_ production can act as a signalling response to Pi to promote better cell migration and adhesion (Figure 13). H_2_O_2_ can also inhibit the Na^+^-dependent Pi transporter and stimulate the low-affinity H^+^-dependent Pi transporter, capturing more extracellular Pi, mainly to supply the energy needs required by the metastatic processes stimulated by Pi (Figure 13).

## 4. Materials and Methods

### 4.1. Materials

All reagents were purchased from high-quality commercial sources, as indicated throughout this section. Radioactive inorganic phosphate (^32^Pi) was obtained from the Brazilian Institute for Nuclear Research. It was purified by extraction as the phosphomolybdate complex with a mixture of 2-butanol/benzene followed by re-extraction to the aqueous phase with ammonium hydroxide and precipitation as the MgNH_4_PO_4_ complex [57]. The distilled water used in the preparation of all solutions was deionized using a Milli-Q system of resins (Millipore Corp., Bedford, MA, USA).

### 4.2. Cells Culture and Pi Incubation

Breast cell lines (MCF10-A, MCF-7, and MDA-MB-231) were grown in routine conditions: 37 °C and 5% CO_2_. A human breast non-tumorigenic epithelial cell line (MCF10-A) was maintained in DMEM-F12 medium, 10% foetal bovine serum (FBS), 10 μg/mL insulin (Merck KGaA, Darmstadt, Germany), 20 ng/mL epidermal growth factor (EGF) (Sigma-Aldrich, St. Louis, MO, USA), 0.5 μg/mL hydrocortisone, pH 7.4 (Sigma-Aldrich, St. Louis, MO, USA), 100 U/mL penicillin and streptomycin (Thermo Fisher, Waltham, MA, USA) [32]. The breast cancer cell lines (MCF-7 and MDA-MB-231) maintained in Iscoves Modified Dulbecco’s Medium (IMDM-LCG Biotechnology, Cotia, São Paulo, Brazil) supplemented with sodium bicarbonate, 10% FBS (Cripion Biotechnology, Andradina, São Paulo, Brazil), 100 U/mL penicillin and streptomycin (Thermo Fisher, Waltham, MA, USA). The pH was adjusted to 7.4 with HCl or NaOH for all culture mediums.

Breast cell lines maintained in IMDM or DMEN-F12 medium are exposed to 1 mM Pi, as stated by the manufacturer. Different concentrations of NaH_2_PO_4_ were added to the regular medium 1 h or 24 h before the assays, as noted in the legends. Before the experiments, cells harvested from the culture medium were washed two times with a buffer consisting of 116 mM NaCl, 5.4 mM KCl, 5.5 mM glucose, 0.8 mM MgCl_2_, and 50 mM HEPES (pH 7.2). Adherent cells were dissociated after incubation at 37 °C and 5% CO_2_ with a trypsin solution (2.5 g/L, pH 7.2, 0.05 mL/cm^2^), and the cell number and protein concentration after Pi treatment were assessed by counting in a Neubauer chamber and the Bradford method, respectively [31,32,33,58].

### 4.3. Pi Transport Assay

MCF-10A, MCF-7 and MDA-MB-231 cells (5 × 10^4^ cells per well) were incubated at 37 °C, 5% CO_2_ atmosphere, for 1 h in a reaction mixture (0.5 mL) containing 116 mM NaCl or choline chloride, 0.1 mM KH_2_PO_4_ or 1 mM KH_2_PO4 (depending on the type of Pi transporter), 5.4 mM KCl, 5.5 mM glucose, 50 mM HEPES (pH 7.2), 0.8 mM MgCl_2_ and 2.5 μCi/nmol ^32^Pi. Reactions were stopped, and cells were washed with 0.5 mL of an ice-cold phosphate buffer saline (PBS) (pH 7.2), and the cells were lysed with 0.5 mL SDS 0.1%. The internalized Pi released by cell lysis was transferred to a filter paper, to then be immersed in scintillation liquid. In parallel, the transport of Pi was carried out at 4 °C as a control (blank values) [31,32].

The high-affinity Na^+^-dependent Pi transport activity was determined using 0.1 mM KH_2_PO_4_ in the reaction mixture, and transport values of Pi in the presence of 116 mM choline chloride were subtracted from the values of transport of Pi in the presence of 116 mM NaCl, thus deriving the NaCl stimulated fraction. To determine the low-affinity H^+^-dependent Pi transport, 1 mM KH_2_PO_4_ was used in the reaction mixture, and NaCl was replaced by 116 mM choline chloride.

### 4.4. Total Intracellular Pi Determination

For total intracellular phosphate measurement, indicated cells (5 × 10^4^ cells/well) were collected by centrifugation and washed with cold water. The cell pellet was resuspended in 200 μL of 1 M H_2_SO_4_ and heated to 95 °C for 20 min [59]. Cell debris was pelleted by centrifugation and supernatants were collected and total intracellular phosphate content was quantified by malachite green assay [60].

### 4.5. Migration Assay

We used a twenty-four-well Corning Transwell© plate with permeable supports and 6.5 mm inserts and polycarbonate membrane pores of 8.0 μm for the migration assays. MCF-10A, MCF-7 and MDA-MB-231 cells (5 × 10^4^ cells/well) were suspended in serum-free medium and maintained at 37 °C and 5% CO_2_ for 24 h. Cells that did not migrate to the lower compartment were removed with a cotton swab. Non-adherent cells were removed with two washes with PBS, and subsequently, 0.5% crystal violet was added to the adherent cells for 5 min, and then they were washed further (2×) with PBS. The washed cells were lysed with acetic acid (1% in ethanol). Cell lysates were read spectrophotometrically at 570 nm. The results are expressed as optical density (OD) at 570 nm [32,61].

### 4.6. Cell Adhesion to Extracelullar Matrix Assay

ECM gel obtained from rat sarcoma cells was diluted in PBS (32 μg/mL) and was used for precoating each well of a 96-well culture plate maintained for 12 h at 4 °C and then blocked with 1 mg/mL BSA. MCF-10A, MCF-7 and MDA-MB-231 cells (2.5 × 10^4^ cells/100 µL) suspended in serum-free medium were added to each well and maintained at 37 °C and 5% CO_2_ for 24 h. After this incubation period, the non-adherent cells were removed by washing twice with PBS. The adherent cells were fixed with 3% paraformaldehyde for 10 min. The fixed cells were then washed with PBS twice, stained with 0.5% crystal violet for 5 min, and washed (2×) with PBS. The washed cells were lysed with acetic acid (1% in ethanol). The content of the cell lysate was read spectrophotometrically at 570 nm. The results are expressed as optical density (OD) at 570 nm [32,61].

### 4.7. Cell Viability

MCF-10A, MCF-7, and MDA-MB-231 cells (1 × 10^4^ cells per well) were seeded in 24-well plates. After 12 h, different Pi concentrations were added to the culture medium, and an additional incubation period of 24 h at 37 °C in a 5% CO_2_ atmosphere was imposed on the cells. Cell viability was assessed using the CellTiter 96^®^ AQueous One Solution Cell Proliferation Assay (MTS) (Promega Corporation, Fitchburg, WI, USA) according to the manufacturer’s instructions. The cell viability also was assessed using LIVE/DEAD™ Viability/Cytotoxicity Kit (1:20; Invitrogen- Thermo Fisher Scientific, Waltham, MA, USA). MDA-MB-231 cell treated by different Pi concentration for 24 h were washed with a reaction mixture (0.5 mL) containing 116 mM NaCl, 5.4 mM KCl, 5.5 mM glucose, 50 mM HEPES (pH 7.2) and 0.8 mM MgCl_2_. Images were acquired with an EVOS fl Fluorescence Microscope from AMG.

### 4.8. H_2_O_2_ Production Assay

The release of H_2_O_2_ produced by the breast cell lines was determined by the Amplex red oxidation (Invitrogen) fluorometric method [62]. MCF10-A, MCF-7 and MDA-MB-231 (1 × 10^6^ cells/mL) were added to a reaction medium containing PBS plus 5 mM glucose, 10 μM Amplex red and 0.1 U/mL horseradish peroxidase (HPR) in a final volume of 0.2 mL. Fluorescence was monitored at excitation and emission wavelengths of 563 ± 5 nm and 587 ± 5 nm for 30 min at 25 °C. H_2_O_2_ concentration was determined using a standard curve with known H_2_O_2_ concentrations.

### 4.9. Mitochondrial Membrane Potential and Mitochondrial ROS Genaration

Mitochondrial membrane potential was analysed in the breast cell lines using the MitoProbe JC-1 assay kit (Molecular Probes - Thermo Fisher Scientific, Waltham, MA, USA). Breast cell lines, MCF10-A, MCF-7, and MDA-MB-231 cells (5 × 10^4^ cells/well) were loaded with 100 nM JC-1 and incubated at 37 °C for 15 min followed by a wash with PBS solution [26]. The ratio of red (540 nm excitation and 590 nm emission) over green (490 nm excitation and 540 nm emission) fluorescence intensity was monitored at 25 °C using a multi-well fluorescence reader.

Mitochondrial superoxide generation was determined using mitoSOX, a red fluorescence dye localized to mitochondria. Cells plated on 18-mm coverslips with 5 × 10^4^ cells/well were treated with Pi for the indicated time and then loaded with mitoSOX 5 μM for 15 min at room temperature [26]. Fluorescence image (excitation/emission 510/580 nm) was obtained by using EVOS fl Fluorescence Microscope from AMG and analyzed by ImageJ software. We performed analysis from more than three independent experiments, more than 5 pictures from each, analyzing most cells in each picture.

### 4.10. Oxygen Consumption Rates

O_2_ consumption rates were evaluated in MDA-MB-231 cells (1 × 10^6^ cells/mL) by high-resolution respirometry (Oxygraph-2K; OROBOROS Instruments, Innsbruck, Austria) in a final volume of 2 mL IMDM medium. Maximum capacity and proton leak were obtained in the presence of 1 µM FCCP and 2.6 nM oligomycin, respectively. To determine the ATP coupled levels, basal levels were subtracted from the values in the presence of 2.6 nM oligomycin. The temperature was maintained at 37 °C, the same as in the growth condition.

### 4.11. ATP Content Determination

The intracellular ATP pool was quantified in the MDA-MB-231 cells using the adenosine 5′-triphosphate (ATP) bioluminescent somatic cell assay kit (Sigma Chemical Co, St. Louis, MO, USA). MDA-MB-231 cells (5 × 10^4^ cells/well) plated in 24-wells were prepared by adding 0.1 mL of somatic cell ATP and releasing reagent in 24-well plates placed ice for 10 min. After that, the cellular extracts were transferred to MTS-11C mini tubes (Axygen Scientific, Union City, CA, USA) containing 0.1 mL of ATP assay mix and mixed for 10 s. The total amount of light emitted was measured with a GloMax Multi JR detection system (Promega Corporation, Fitchburg, WI, USA). The total intracellular ATP concentration per cell number was calculated using a standard ATP curve prepared and analysed in each experiment.

### 4.12. Protein Kinase C Activity Assay

MDA-MB-231 cells (5 × 10^4^ cells/well) were washed twice in 116mM NaCl, 5.4 mM KCl, 5.5 mM glucose, 50 mM HEPES (pH 7.2), 0.8 mM MgCl_2_ and lysed in HEPES 50 mM Mes-buffer plus Triton X-100 0.1%. PKC activity was assayed in the presence of 4 mM Hepes-Tris, pH 7.0, 0.4 mM MgCl_2_, 1 mM CaCl_2_, 0.36 mg/μL Neurogranin (specific substrate for PKC), 25 nM ATP and 50 μg of lysed cells in a final volume of 50 μL in MTS-11C mini tubes (Axygen Scientific, Union City, CA, USA). The reaction was triggered by adding 40 μL of the Kinase-Glo luminescent kit, and after 10 min at 37 °C were placed in a luminometer (Promega Multi Glomax Junior).

### 4.13. Western Blotting

For Western blotting detection, MDA-MB-231 cells were lysed with 1 mL RIPA buffer for 30 min. Then, homogenates (100 µg) were separated by 12% or 7.5% SDS-PAGE (7.5% for E-cadherin analysis) and transferred to nitrocellulose membranes (Merck Millipore, Burlington, MA, USA). Non-specific binding was prevented by incubating the membranes with 5% non-fat milk in TBS for 1h. Then, the membranes were probed overnight at 4 °C with the corresponding primary antibody (dilution 1:500 for PKC-α, PKC-ε, PKC-λ, PKC-ζ, snail, GAPDH and dilution 1:1000 for IkBα, E-cadherin or vimentin). After this incubation, membranes were washed three times with TBS containing 0.1% Tween 20, and detected using a correspondent anti-rabbit or anti-mouse (for vimentin or GAPDH) HRP-conjugated IgG secondary antibody. Primary antibodies were from Sigma Chemical Co.

### 4.14. Statistical Analysis

In all cases, at least three independent experiments were performed in triplicate. The values shown in all experiments represent the mean ± SE. The significance of any difference in each parameter among the groups was evaluated by one-way analysis of variance (ANOVA) and Tukey’s multiple comparisons test, and the criteria for statistical significance were: *, *p* < 0.05. All statistical analyses were performed using Prism 6.0 software (GraphPad Software, San Diego, CA, USA).

## Data Availability

The data that support the findings of this study are available from the corresponding author upon reasonable request.

## Supplementary Materials

Supplementary materials can be found at https://www.mdpi.com/article/10.3390/ijms221810096/s1.
