# Supplementary material for: Hydrogen Peroxide Generation as an Underlying Response to High Extracellular Inorganic Phosphate (Pi) in Breast Cancer Cells
Source: Int J Mol Sci. 2021 Sep 18;22(18):10096. doi: 10.3390/ijms221810096 (PMC8468810; doi:10.3390/ijms221810096)
Supplement: Supplementary file 1 [file ijms-22-10096-s001.zip › ijms-1214955-supplementary.pdf]

Supplementary Files

Material and Methods

Quantitative PCR

Total RNA was extracted using TRIzol Reagent (Thermo Fisher Scientific) as described by manufacturer's manual from MDA-MB-231, MCF-7 or MCF-10 cells. After treatment of RNA with DNase I, 1 µg of total RNA was submitted to reverse transcription using the High-Capacity cDNA reverse transcription kit (Thermo Fisher Scientific). For qPCR assay, 500 ng/ml cDNA was used per well, using a FastStart Master SYBR Green I Kit (Roche). qPCR was carried in StepONEPlus Real Time PCR System (Applied Biosystems). The primers for amplification are shown in Table S1. Gene expression data were normalized to an endogenous reference β-actin (ACTB) as previously described (Lacerda-Abreu et al., 2019 and 2018) and according to the manufacturer's instructions.

Table S1. Primers sequences

Sequence name	Sequence	Reference
NaPi 1_For	CGTATGTCTTCTCTGGTTCGTTCTG	[31].
NaPi 1_Rev	CGTAAAACTACCAGTGGAAATAGCCC	[31].
NaPi-IIa_For	GTGGCCTCCTTCAACATCCAT	[31].
NaPi-IIa_Rev	CTGTAAGGAGTCTGGGTGGC	[31].
NaPi-IIb_For	CCCAGCTTATAGTGGAGAGCTTC	[31].
NaPi-IIb_Rev	GCACCAAATCTTGACAAGACTCTTG	[31-32].
NaPi-IIc_For	GAATTTCAGAGGGCTTTCAGCG	[31].
NaPi-IIc_Rev	GAGTCCAACTGCACGATGAGG	[31].
PiT1_For	CCTAATGGTTTGCGAGCTTTGC	This work
PiT1_Rev	GAACCAGACGATAAGGGCACAG	This work
PiT2_For	GATGACAGCACCATCCCG	This work
PiT2_Rev	GGTACACATGACCGTCGCTC	This work
ACTB_For	TGACGTGGACATCCGCAAAG	[31-32].
ACTB_Rev	CTGGAAGGTGGACAGCGAGG	[31-32].

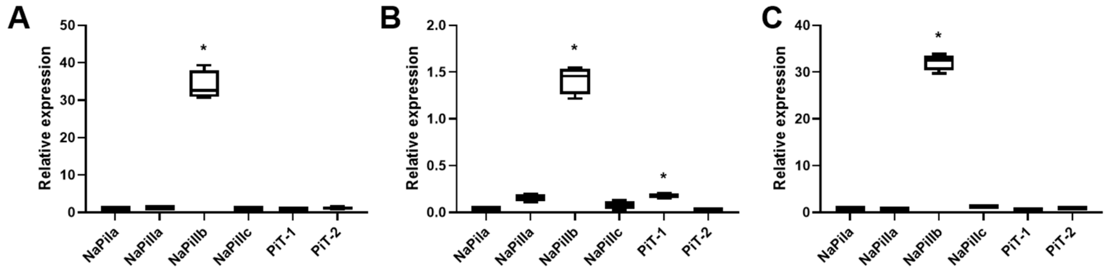

Figure S1: Comparison of transcription level of Pi transporters in MCF-10A, MCF-7 and MDA-MB-231 cells. Total RNA was purified from the three different cell lineages, as described in Material & Methods, and qPCR was carried. Gene expression data were normalized to an endogenous reference β-actin (ACTB). The results are the means ± SE of 4 experiments with different cell suspensions. Asterisks mark significant differences ($p \leq 0.05$) from the NaPiIa of MCF-10 cells (A), as determined by One-Way analysis of variance (ANOVA), using Dunnett's multiple comparisons test.

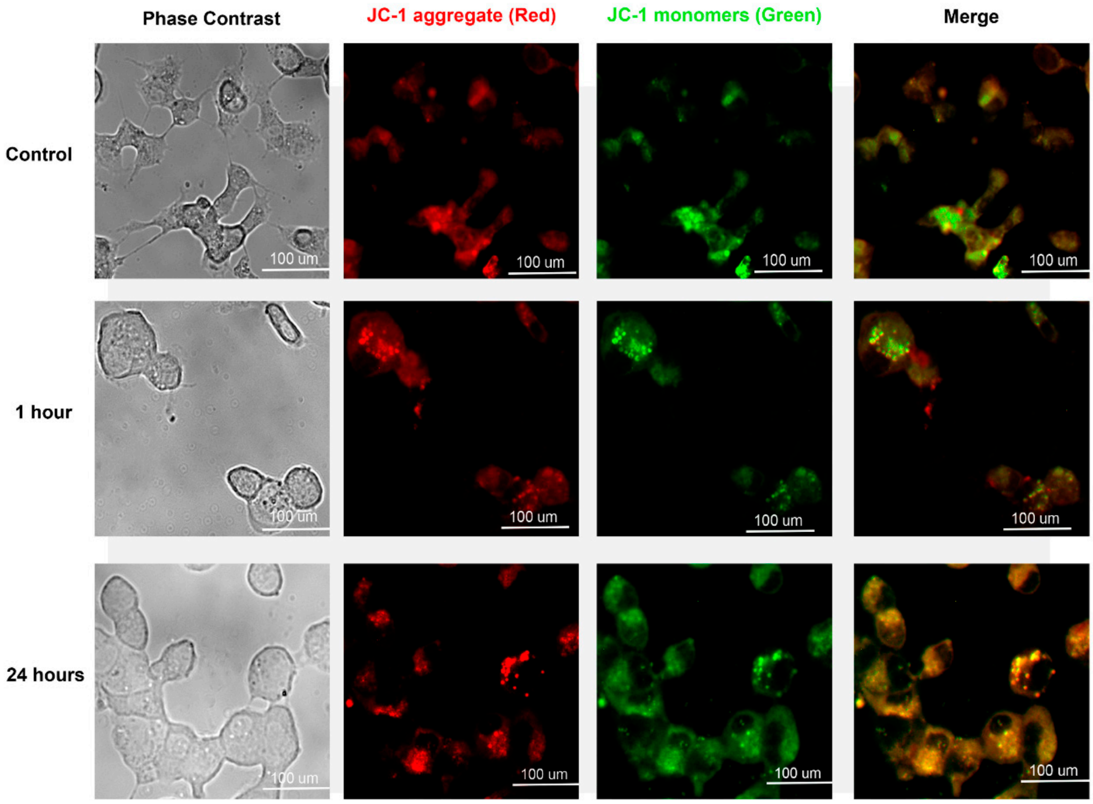

Figure S2: Mitochondrial membrane potential (ΔΨm) by JC-1 staining MDA-MB-231 cells treated with 1 mM Pi and 8 mM Pi (1 or 24 hours). Representative phase contrast images (left panels) and JC-1 fluorescence images (right panels). MDA-MB-231 mitochondrial membrane potential was measured by JC-1, an indicator of mitochondrial function, red fluorescence represents the dependent aggregate of mitochondrial potential JC-1, after depolarization, green fluorescence remains, indicating JC- 1 monomeric. The ratio of red/green fluorescence was markedly higher in MDA-MB-231 treated with 8 mM Pi for 1 h. MDA-MB-231 treated by 8 mM Pi for 24 h showed the same red/green fluorescence ratio compared with control cells (1 mM Pi). Slides were observed in EVOS fl Fluorescence Microscope from AMGe and images were processed using Adobe Photoshop software.

Table S2. Predicted phosphorylation sites with scores ≥0.60 and specific for PKC in the primary sequences of NaPiIIb isoform 1 and 2

NaPiIIb isoform 1		NaPiIIb isoform 2	
Amino acid	Score	Amino acid	Score
Thr^{37}	0.718	Thr^{35}	0.871
Thr^{113}	0.638	Thr^{57}	0.654
Thr^{136}	0.654	Ser^{82}	0.648
Ser^{161}	0.648	Thr^{86}	0.650
Thr^{165}	0.650	Thr^{153}	0.717
Thr^{232}	0.717	Thr^{163}	0.786
Thr^{242}	0.786	Thr^{176}	0.916
Thr^{255}	0.916	Thr^{217}	0.686
Thr^{296}	0.686	Thr^{304}	0.831
Thr^{383}	0.831	Thr^{306}	0.725
Thr^{385}	0.725	Thr^{336}	0.754
Thr^{415}	0.754	Thr^{341}	0.783
Thr^{420}	0.783	Ser^{388}	0.846
Thr^{467}	0.846	Thr^{396}	0.926
Thr^{475}	0.926	Thr^{426}	0.641
Thr^{505}	0.641	Ser^{431}	0.662
Ser^{510}	0.662		

Analysis of phosphorylatable sites for NaPiIIb isoform 1 (GenBank: O95436) and NaPiIIb isoform 2 (GenBank: O95436.2) using NetPhosK 3.1 prediction software
